# Advanced Static and Dynamic Fluorescence Microscopy Techniques to Investigate Drug Delivery Systems

**DOI:** 10.3390/pharmaceutics13060861

**Published:** 2021-06-11

**Authors:** Jacopo Cardellini, Arianna Balestri, Costanza Montis, Debora Berti

**Affiliations:** Department of Chemistry “Ugo Schiff” and CSGI, University of Florence, Via della Lastruccia 3–13, 50019 Florence, Italy; jacopo.cardellini@unifi.it (J.C.); arianna.balestri@unifi.it (A.B.); debora.berti@unifi.it (D.B.)

**Keywords:** drug delivery, nanoscopy, super-resolution fluorescence microscopy, STED, STORM, PALM, TIRF, light-sheet microscopy, FCS, particle tracking, FRAP

## Abstract

In the past decade(s), fluorescence microscopy and laser scanning confocal microscopy (LSCM) have been widely employed to investigate biological and biomimetic systems for pharmaceutical applications, to determine the localization of drugs in tissues or entire organisms or the extent of their cellular uptake (in vitro). However, the diffraction limit of light, which limits the resolution to hundreds of nanometers, has for long time restricted the extent and quality of information and insight achievable through these techniques. The advent of super-resolution microscopic techniques, recognized with the 2014 Nobel prize in Chemistry, revolutionized the field thanks to the possibility to achieve nanometric resolution, i.e., the typical scale length of chemical and biological phenomena. Since then, fluorescence microscopy-related techniques have acquired renewed interest for the scientific community, both from the perspective of instrument/techniques development and from the perspective of the advanced scientific applications. In this contribution we will review the application of these techniques to the field of drug delivery, discussing how the latest advancements of static and dynamic methodologies have tremendously expanded the experimental opportunities for the characterization of drug delivery systems and for the understanding of their behaviour in biologically relevant environments.

## 1. Introduction

The development of nanomaterials for the delivery and controlled release of drugs to a selected, specific biological target has been, for many years, one of the core areas of nanomedicine research. Indeed, the design and preparation of smart nanocarriers—with encoded targeting and/or controlled release abilities, multifunctional therapeutic or combined therapeutic/diagnostic properties, and responsivity to diverse stimuli—have reached, over the years, unpredictable achievements [1]. Significant contributions to the accomplishments in the design/synthesis of complex drug delivery systems (DDSs) have resulted from the continuous theoretical progression and the improvements of fundamental knowledge on nanomaterials, as well as from the technical advancements of experimental techniques for nanomaterials characterizations. However, despite the successful realization of countless carriers for the targeted delivery of therapeutics, only a few of them—aside from the very recent lipid vector-based vaccines against COVID-19 [2]—are FDA- or EMA-approved [3]. The gap between the synthesis of the DDSs and their full translation into medical practice is related to poor understandings of their behavior in biological environments [4,5]. In this respect, many efforts are currently devoted to the improvement of knowledge regarding the fate of nanomaterials designed for biomedical applications in living organisms, and of their behavior within biological fluids and with biologically relevant interfaces, such as cell membranes [5,6,7,8,9].

In this framework, fluorescence microscopy and laser scanning confocal microscopy (LSCM) are key experimental techniques to unravel the behavior of nanomaterials designed for pharmaceutical applications in biological environments [10,11,12,13]. Compared to other imaging techniques, in fluorescence microscopy the signal (and, consequently, the contrast) is provided by fluorescence (or autofluorescence); therefore, choosing the appropriate fluorescent probes allows for the highlighting of specific characteristics of the sample (as hydrophobic/hydrophilic regions), and/or the labeling of selected groups of the sample of interest, in an extremely tunable and variable manner. Fluorescence (confocal) microscopy is, for instance, applied to determine the extent of cellular uptake of fluorescently labeled nanocarriers or active principles and/or the occurrence of cytotoxic effects—both relevant pieces of information for enabling a careful evaluation of the efficacy and potential risks associated with the administration of a DDS. Compared to standard fluorescence microscopy, the confocal setup increases the effective signal-to-noise ratio, thanks to the presence of two pinholes. One of the pinholes reduces the spatial dimension of the excitation beam, while the other removes the out-of-focus emitted light. The advent of confocal microscopy has represented a major advancement in fluorescence microscopy, providing the ability to track the localization of DDSs in complex biological media and to optically reconstruct the three-dimensional space around them with extremely low out-of-focus noise and improved spatial resolution.

In addition, aside from imaging techniques, which provide the ability to unravel the localization of DDSs in complex environments, several fluorescence microscopy-related techniques have been developed and refined over the years, thus allowing for informational gain related to the dynamics of DDSs (i.e., their diffusion modes and rates). Fluorescence recovery after photobleaching (FRAP), fluorescence correlation spectroscopy (FCS), and particle tracking (PT) can provide (at different timescales, on different systems, and with different theoretical frameworks and experimental setups) information on the dynamics of DDSs, or on the modification of the typical dynamics of biological environments in response to the interaction with a DDS. Crucial information on DDS characteristics and behaviors in a complex biological environment can be obtained through these techniques, as the internalization mode of a nanocarrier inside a cell lumen (i.e., the specific internalization route, as well as its internalization form, as an assembled or disassembled entity), its adhesion to a biological interface, and its interaction with relevant biomolecules present within biological fluids [14,15,16,17].

Despite the extensive application of LSCM in the characterization of biological systems and nanostructured DDSs, a clear limitation is represented by the achievable spatial resolution, which is far from that of electron microscopes. Indeed, typical sizes of nanostructured DDSs are within the range of a few–a few tens or hundreds of nanometers, which are also the typical length scales of DDS interactions with the surrounding environment, while the resolution limits of LSCM are 200–300 nm in the xy plane and 500–700 nm in the axial direction (depending on the optical setup and on the wavelength of the laser line). Due to this inherent physical limit imposed by the diffraction limit of light, the information obtained through LSCM on the characteristics of nanostructured DDSs, as well as on their impact on subcellular processes or interaction with biological interfaces, is limited.

In recent years, different methods have been developed to break the physical diffraction limit of light, approaching the typical nanometric resolution of electron microscopy. From the recognition of the potential groundbreaking impact of super-resolution imaging, with the 2014 Nobel prize in Chemistry, different nanoscopy techniques have been implemented on commercial microscopes, and are constantly refined to expand their applicability. Driven by this new hype around super-resolution microscopy, a general renewed interest has grown on advanced fluorescence microscopy techniques, which now offer multiple options and new opportunities for the characterization of nanostructured objects as DDSs, and of their behaviors (in terms of static localization and dynamic motion) in biological environments, on a nanometric length scale.

In this review, we summarize the major recent advancements of fluorescence microscopy-related techniques, in view of their impact in the field of drug delivery. Specifically, in Section 2, we consider advanced imaging techniques, with particular focus on super-resolution techniques (Section 2.1) and techniques designed to investigate thin layers/surfaces and thick samples (Section 2.2); in Section 3, we revise the main fluorescence microscopy-related techniques to investigate the dynamics of DDSs, with particular focus on the impact of the recent advent of super-resolution imaging on these techniques in the field of drug delivery. For the different experimental techniques, the opportunities offered by these novel tools, in relation both to DDS characterization and to the description of DDS behavior in biological environments, are summarized, highlighting the current opportunities and potentialities of static and dynamic advanced fluorescence microscopy-related techniques within drug delivery research.

## 2. Advanced Imaging of Drug Delivery Systems

The main application of advanced fluorescence microscopy-based techniques to the investigation of DDSs is the determination of the localization of DDSs in vitro and in vivo, which is a key issue, for instance, to: (i) understand the behavior of DDSs with biological interfaces/barriers and in biological media; (ii) evaluate the degree of cell uptake and understand cell uptake routes in vitro; and (iii) determine the extent of DDS accumulation in selected tissues. In these respects, the main advantage represented by super-resolution techniques is the possibility to accurately determine the localization of DDSs in complex biological samples. Clearly, together with opportunities, several challenges arise. To mention a few: some super-resolution techniques necessitate the use of powerful laser sources (in particular, STED), which on one hand requires a tailored design of optimized photostable fluorescent probes, while on the other hand, might determine the photodamage and phototoxicity of biological samples in cases of long exposure [18]; biological samples are highly complex, therefore it is necessary to establish the efficacy/quantum yield and/or the possible inactivation of the dye in the environment of interest [19,20,21]; the thickness of the biological sample of interest challenged by the DDSs strongly varies from the five nanometers of a synthetic cell membrane model layered on a converglass [22,23,24], to the tens of micrometers of a eukaryotic cell, to the millimeters of a tissue specimen, thus requiring different tailored solutions [25]; in vivo imaging is extremely challenging and requires a specific setup and dedicated protocols (see, for instance, [26]), including the use of (relatively) biocompatible fluorescent dyes, which have to be carefully chosen when designing the experiment (in this respect, in live-cell STED microscopy, there is a common use of genetically encoded markers, such as fluorescent proteins [27,28]). In the following paragraph, the main advanced fluorescence microscopy-related imaging techniques of interest in the investigation of DDSs will be reviewed. In Section 2.1, the main techniques for super-resolution imaging (namely, stimulated emission depletion microscopy (STED), stochastic optical reconstruction microscopy (STORM), and photoactivated localization microscopy (PALM)) will be briefly presented, and their application to the field of drug delivery will be discussed, with a particular focus on the recent advancements and the many diverse opportunities offered by the techniques for this research area. In Section 2.2, the main recent applications and advancements of light-sheet fluorescence microscopy (LSFM) and total internal reflection fluorescence (TIRF) will be reviewed, particularly in relation to super-resolution imaging and sample thickness.

### 2.1. Super-Resolution Imaging—STED, PALM, STORM

**Figure 1:**
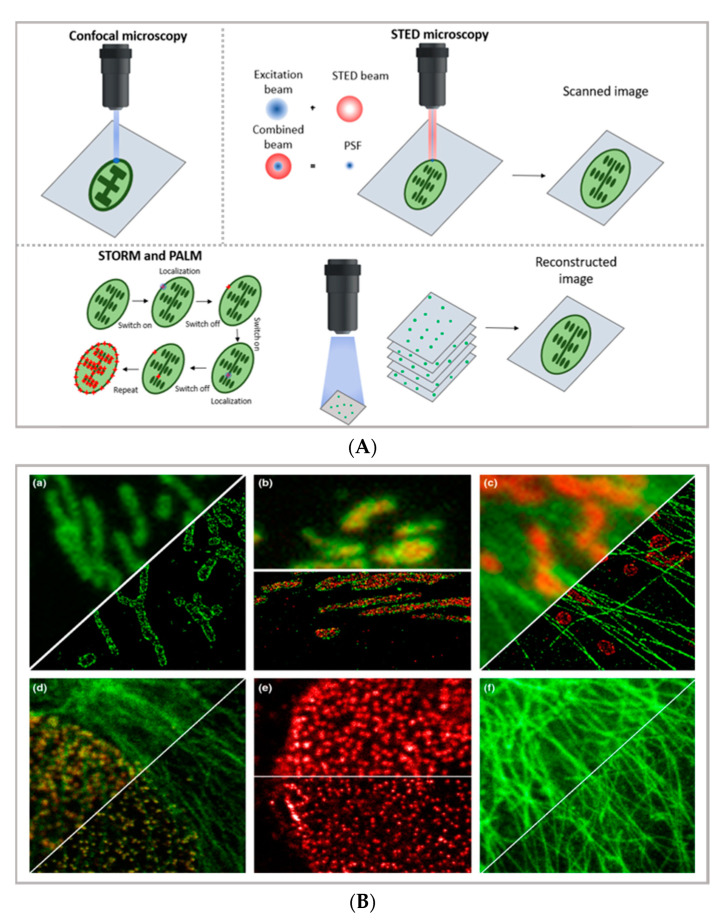

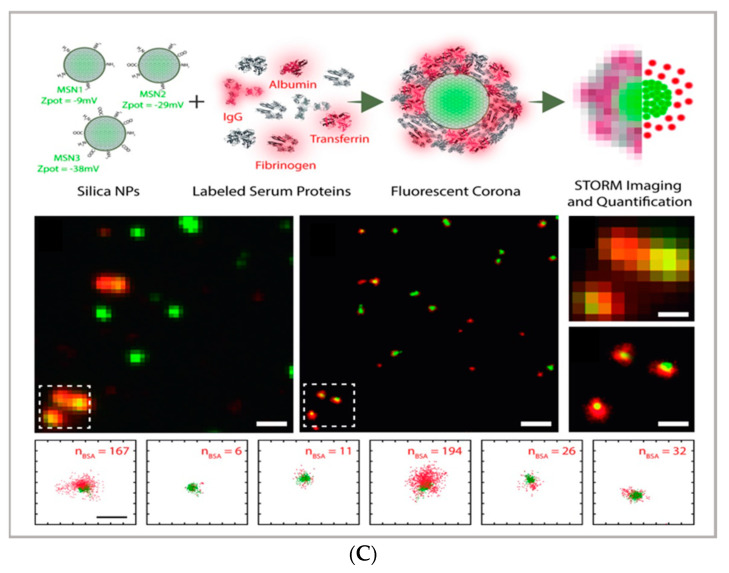
(**A**) comparison between the image acquisition/reconstruction modes of different techniques. Laser scanning confocal microscopy (LSCM) is based on a diffraction-limited point acquisition, which is scanned across the specimen through the scan-head to reconstruct the full image. Stimulated emission depletion microscopy (STED) acquisition relies on the combination of two diffraction-limited laser beams (an excitation beam and a donut-shaped depletion beam), whose combination produces a virtually unlimited decrease in the size of the point spread function; the image acquisition/reconstruction is obtained, similarly to LSCM, through a non-diffraction limited point acquisition, which is scanned across the specimen through the scan-head, to reconstruct the full image. STORM/PALM techniques rely on the employment of low concentrations of photo-switchable probes excited at a low intensity; the stochastic activation of single fluorophores allows for the determination of their precise localization, whereas repeated on-off cycles allow for the random activation of all the fluorophores in the specimen, and the combination of the cycles allows for the reconstruction of the high-resolution image [32]. (**B**) examples of high-resolution STORM and STED images compared to lower resolution LSCM images acquired for the same sample: (**a**–**c**) STORM images of BS-C-1 cells ((**a**,**b**) mitochondrial proteins Tom20 (green) and ATP Synthase (red), and (**c**) mitochondria protein Tom20 (red) and microtubules (green)); (**d**–**f**) multi-color STED images of PtK2 cells. All images are 10 × 10 µm in size (adapted with permission from [31] copyright (2015) WILEY-VCH Verlag GmbH & Co. KGaA, Weinheim, Germany). (**C**) examples of super-resolution techniques applied to drug delivery research (adapted with permission from [33]. Scale bar 200 nm; copyright (2017) WILEY-VCH Verlag GmbH & Co. KGaA, Weinheim, Germany).

In the latest years, the advent of super-resolution microscopy (SRM) has represented a game-changer in the characterization of the behavior of DDSs in biological systems—and more in general, in biomedical research—holding the promise to combine the inherent advantages of confocal microscopy (i.e., the possibility of directly visualizing biological samples without the need for complex data analysis, the non-invasive and biocompatible nature of the technique, and the possibility of highlighting specific areas of even highly complex biological samples through an appropriate selection of the fluorescent tags) with an extremely high resolution, thus approaching the limits of electron microscopy. A thorough description of the different SRM techniques is beyond the scope of this review (the reader is advised to refer to specific reviews, such as [28,29,30]); however, it is useful to briefly summarize the basic principles and differences of the two main experimental approaches adopted—reversible saturable optical fluorescence transitions (RESOLFT) microscopy on one side (for which the main representative technique is STED) and single-molecule localization microscopy (SMLM) on the other side (for which the main representative techniques are STORM and PALM)—in order to discuss the specific limitations and opportunities provided by diverse SRM techniques in the field of drug delivery (see Figure 1B) [31].

STED imaging, first conceptualized in 1994 by Hell et al. [34], enables the collection of images with a theoretically unlimited resolution. The basic principle of STED (briefly schematized in Figure 1A) is to combine two diffraction-limited beams (an excitation beam and a donut-shaped depletion beam) to obtain a virtually unlimited decrease in the size of the point spread function (PSF). STED setup is based on two pulsed laser beams: the excitation beam brings all fluorophores present in the diffraction-limited spot to the excited state; the second, delayed, donut-shaped depletion beam suppresses the periphery’s fluorescence of the illuminated spot. Through this setup, it is currently possible to achieve a 30 nm lateral resolution and a below 100 nm axial resolution [28]. Though the STED process was first theorized two decades ago, its possibilities are still far from being fully explored. Ideally, the STED approach enables the combination of the high resolution typical of electron microscopy with the typical simpler and non-invasive sample preparation of fluorescence imaging. Moreover, the technique allows for in vivo imaging, as well as the study of subcellular interaction mechanisms and the simultaneous detection of multiple systems exploiting distinct targeting probes. STED image acquisition is relatively fast (currently, the acquisition rate of an image is limited by the scanner, rather than by the STED process); however, it requires high source power, which might be carefully considered in the application of the technique to living organisms and/or long live acquisitions of living cells.

A different approach to achieving the super-resolution adopted in super-resolved, single-molecule localization microscopy is based on the localization of the fluorophores’ position under diffraction limit accuracy, which is completed by determining the centroid of the emitting spot. In particular, as briefly schematized in Figure 1A,C, stochastic optical reconstruction microscopy (STORM) and photoactivated localization microscopy (PALM) rely on the employment of photo-switchable/photo-activatable probes, which are dispersed within the sample in a rather diluted amount. When illuminated by a relatively low-intensity source, some dyes are stochastically activated and emit photons before decaying back to the dark state. If the activated dyes are sufficiently separated from each other, it is possible to exploit the stochastic photon emission events to precisely evaluate the central position of the emitting spots. In SMLM, the high-resolved image can be reconstructed through performing multiple cycles of activation and deactivation of the fluorophores, collecting multiple images, and combining the localized positions of molecules to reconstruct the final image—finally providing the high-resolution, even subcellular, details of biological specimens. With STORM/PALM techniques, higher spatial resolution (with lower illumination intensity than STED) is obtained, with relatively cost-effective implementations of conventional wide-field microscopes; however, a longer acquisition time and advanced image processing are required, as well as a careful choice of fluorescent probes. Conversely, fast image acquisition, without requiring complex data processing, can be achieved with STED. As a general rule of thumb in drug delivery research, SMLM techniques—with lower acquisition intensity and a slower acquisition time—can be profitably employed in the detection of relatively slow processes (such as DDS uptake and localization in living cells) with slightly higher resolution and a lower risk for phototoxicity than STED, while STED is the technique of choice for faster dynamics (as discussed in Section 3). In addition, the single-molecule detection nature of STORM and PALM, which is inherently required to reconstruct the high-resolution image, can be exploited for quantitative imaging; some examples are reported below. A more detailed comparison of the super-resolution techniques can be found in other works [31,35].

From an applicative perspective, all SRM techniques represent powerful tools in cellular biology and pharmaceutical application. Each of the techniques provides direct visualization of engineered DDSs in vitro and in vivo in cells, tissues, and living organisms, and thus, direct proof of their efficacy in reaching their biological target. However, they can also represent valuable tools in characterizing complex, nanostructured DDSs; complementing the structural, colloidal, and interfacial information obtained through common characterization techniques applied to nanomaterials—such as scattering (i.e., dynamic light scattering, small-angle X-ray, and neutron scattering), surface techniques (i.e., quartz crystal microbalance with dissipation monitoring and atomic force microscopy), and electron microscopy. For instance, STORM microscopy has been recently applied to investigate the formation of the protein corona coating silica nanoparticles, allowing for the quantification of the dynamic inhomogeneities in the protein corona layer [33] and thus opening up the perspective to investigate the behavior of DDSs within relevant biological fluids. A similar multicolor approach has also been applied to investigate the localization and exchanges of monomers within dynamic supramolecular polymer systems, showing a possibility to exploit super-resolution microscopy to investigate the inner dynamics of multicomponent soft matter systems [36]. A significant opportunity in the design/synthesis of nanocarriers for drug delivery is the potential ability to functionalize the surface with targeting moieties and/or other bioactive principles; however, it is often challenging to quantitatively evaluate the functionalization extent. In this respect, the single-molecule nature of SMLM [37] can be exploited to estimate the number of active functionalities on a particle surface [3,38]. For instance, Belfiore et al. quantitatively estimated the amount of targeting moieties (specifically, plasminogen activator inhibitor-2 (PAI-2) and trastuzumab (TZ, Herceptin^®^) targeting cancer cell surface biomarkers) on functionalized liposomes [39], showing how this experimental/analytical approach can be applied for the characterization of a multifunctional drug delivery system.

Aside from these examples where SRM is applied to characterize the DDSs themselves, the key contribution of SRM to drug delivery research is represented by the possibility to achieve a direct visualization and the sub-diffraction, nanometric localization of DDSs in biological environments. In this respect, a general issue in SRM is in the choice of suitable fluorescent probes to appropriately label both the nanocarriers/active principles and cellular/subcellular compartments. This issue also applies to fluorescence microscopy; however, in super-resolution techniques, the photophysical properties of the dyes, such as photostability, brightness, and the ability to control the ON/OFF switch of excited states, are determinant factors in the formation of super-resolved images, themselves. Therefore, in the latest years, researchers have focused on synthesizing/developing suitable fluorescent probes, especially for the appropriate labeling of complex cellular environments [18,40,41,42,43,44,45]. Recently, the development of nanoparticles (of an inorganic, organic, or biogenic nature) for super-resolution imaging has gained increased interest, due to the possibility of combining the new opportunities offered by super-resolution, in terms of diagnostics (with specific characteristics of nanoparticles), as therapeutic agents, or as biosensing probes [46,47]. In this framework, Shang et al. [3] report on the synthesis of dye-labeled transferrin protein-based NPs with elevated photostability for super-resolution imaging in live-cell nanoscopy, thus combining the properties of transferrin-based nanoparticles (as biocompatible carriers with cancer cells’ targeting properties) with the photostability of the Atto647N dye. Another example, presented by He et al., shows how small blinking, single-layer graphene nanosheets can serve both as nanoscopy fluorophores and as drug-bearing nanocarriers [48,49].

A relevant contribution of super-resolution microscopy to drug delivery research is the potential ability to localize a DDS inside cells with high spatial resolution, thus allowing not only for the evaluation of its cell uptake and cell uptake extent, but also its proximity to—and, therefore, its interaction with—specific cellular compartments. In this respect, super-resolution techniques have been applied to understand the endocytic pathways of nanocarriers. For instance, the internalization of cancer-derived exosomes in HeLa cells was detected using PALM/STORM imaging, by revealing the colocalization inside lysosomes [50], while the direct visualization of intracellular mechanisms for gene delivery has been reported for both siRNA-complexes and polyplexes, carrying plasmid DNA and interacting with cells, through STORM [51,52]. However, for relatively large organelles, such as mitochondria, regular LSCM can also be used to achieve such tasks [53,54].

Super-resolution techniques have also been successfully applied to the investigation of the interaction between nanocarriers and DDSs with relevant biological barriers, such as the dermal barrier and the blood–brain barrier. Specifically, Brewer et al. [55], by applying the nanoscopic resolution of the STED, investigated molecular penetration routes through the stratum corneum, while Lammers et al. [56] applied STED to study the accumulation of fluorescent polymeric and lipid nanocarriers in the brain cells of healthy mice.

### 2.2. Imaging Thin Layers and Thick Samples: TIRF, LSFM

As already mentioned in the Section 1, the thickness of the samples of potential interest in drug delivery research can strongly vary; for instance, studies on the interaction of nanocarriers with four–five nanometers-thick supported lipid bilayers require the employment of surface techniques, while investigations on tissue slices of some millimeters thickness require the employment of techniques with the ability to deeply penetrate inside the specimen. In both cases, the super-resolution techniques described in the previous paragraph might not be the best choice.

**Figure 2:**
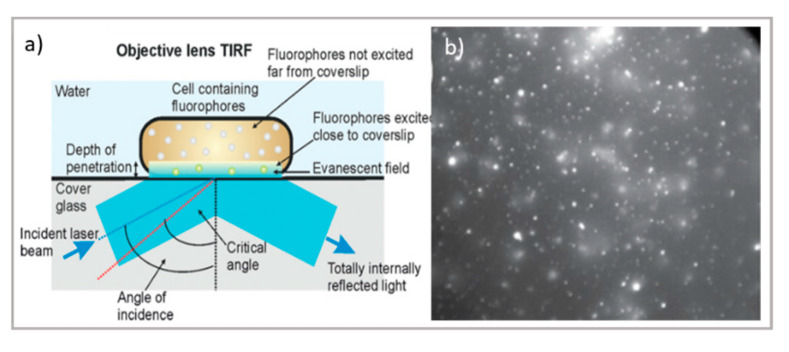

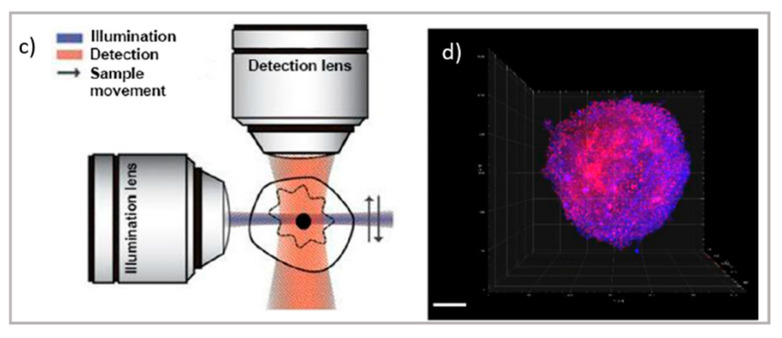
Top: schematic illustration of (**a**) TIRF microscope [66] and TIRF micrographs; (**b**) SLBs formed on planar silica with auto-fluorescent felodipine. Scale bar = 20 mm (adapted with permission from [61]; copyright (2020), the authors; Angewandte Chemie published by Wiley-VCH GmbH); bottom: schematic illustration of (**c**) the LSFM microscope and tomography; (**d**) LSFM of large multicellular tumor spheroids (Scale bar 100 µm); readapted from [63] (copyright (2019), with permission from Elsevier).

Concerning thin samples, total internal reflection fluorescence (TIRF) microscopy is characterized by a relatively simple experimental setup—allowing for thin samples to reach a high resolution close to that of STED while applying a localized illumination with minimum time and light power exposure—making it particularly suitable for live-cell imaging. As briefly schematized in Figure 2a, TIRF exploits the total internal reflection process at the glass–water interface to produce an evanescent field protruding 100–200 nm through the sample, making the technique extremely sensitive at the very interface between the glass and the specimen. Lateral and axial resolutions range from 50 to 100 nm [57], therefore making them comparable with super-resolution techniques, though with a much simpler experimental setup. In addition, this thin, excited section extends the lifetime of the cellular specimens, reducing the photobleaching and phototoxic damage generally induced by the high power of the incident light (for instance, necessary for STED). For this reason, TIRF is the most appropriate technique for the characterization of biological processes occurring close to the cellular membrane, such as transmembrane protein-mediated endocytosis and exocytosis, which is crucial for drug delivery [58,59]. Other applications of TIRF in drug delivery research include the study of the interaction of nanocarriers/nanomaterials designed for nanomedicine applications with synthetic biomimetic membranes. For instance, Conn et al. investigated the fusion kinetics of lipid nanocarriers with model membranes and cells through TIRF, thus extending the knowledge of the interaction between drug vectors and cellular barriers [60], while Hook et al. recently studied the kinetics of drug permeation through a model membrane by combining TIRF and fluorescence recovery after photobleaching (FRAP) [61].

Concerning thick samples, LSCM and super-resolution microscopies are generally limited to 60–80 µm of depth. Thick samples, as tissue slices, can be investigated through light-sheet-based fluorescent microscopy (LSFM) [62]. The LSFM setup relies on the sectioning of sample layers with a sheet illumination (see Figure 2a). Through this setup, thick samples can be imaged at various depths, increasing the 3D visualization of samples up to the millimeter scale. In drug delivery research, LSFM has been applied to the investigation of drug penetration in 3D cell culture models, which is often challenging due to the difficulty in the long-depth collection of large samples. Recently, LSFM has been applied for the permeation analysis of model drugs in multicellular tumor spheroids [25,63]. A major improvement of light-sheet microscopy is its combination with STED, allowing for the strong improvement in both axial and lateral resolution while maintaining the typical penetration depth capabilities of light-sheet microscopy [64,65].

## 3. Dynamics of Drug Delivery System

**Figure 3:**
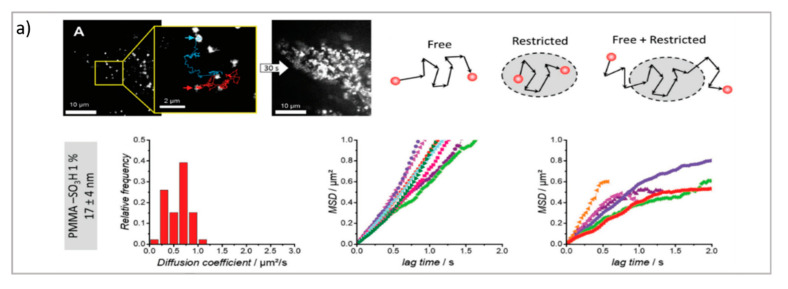

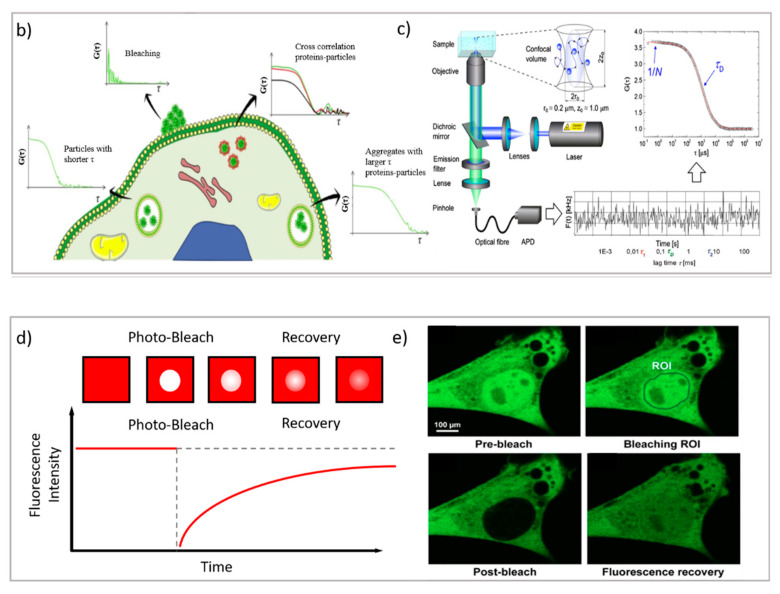
(**a**) PT technique: diffusion of NPs in the cytosol studied using single-particle fluorescence tracking and three schematic illustrations of intracellular diffusion—free, restricted, and a combination of free and restricted diffusion (adapted with permission from [67]; copyright (2018) WILEY-VCH Verlag GmbH & Co. KGaA, Weinheim, Germany). (**b**,**c**) FCS technique: **(b)** examples of FCS curves measured in different cellular compartments reprinted from [16] (copyright (2019), with permission from Elsevier), (**c**) a schematic illustration of FCS set-up reprinted from [68] (copyright (2012), with permission from Elsevier). (**d**,**e**) FRAP technique: (**d**) basic representation of the FRAP approach [69], (**e**) example of a FRAP experiment on a myoblast cell line (myo3), before and after bleaching [70].

As reviewed in the previous section, thanks to the advancements of fluorescence microscopy, it is now possible to determine the localization of drug delivery with nanometric resolution. This undoubtedly represents an opportunity to understand the fate of DDSs in living organisms. Another opportunity offered by fluorescence microscopy and laser scanning confocal microscopy-related techniques is to determine not only the localization of nanosystems or drug delivery systems but also their dynamics. Techniques such as particle tracking (PT), fluorescence correlation spectroscopy (FCS), and fluorescence recovery after photobleaching (FRAP), which basic principles are summarized in Figure 3, can be useful to thoroughly determine the behavior of the DDSs in vitro and in vivo. In particular, these techniques can be used to: (i) characterize the cellular uptake of DDSs (which is the internalization pathway; if the DDS is internalized by cells as a whole or in a disaggregated form); (ii) characterize the binding of the DDS to relevant biomolecules; (iii) characterize the motion of the DDS in bio-relevant fluids; and (iv) characterize the interaction of the DDS with biomembranes. In addition, these techniques have also been profitably exploited to characterize the DDS itself, thus providing additional relevant information with respect to the most common physical chemistry techniques. In recent years, the diverse techniques have also taken advantage from the new advancements of fluorescence microscopy toward super-resolution, allowing the limit of dynamic characterization of DDSs to be pushed toward a nanometric length scale. In the following paragraph, these techniques will be reviewed, and the opportunities provided by each technique for the field of drug delivery will be highlighted.

### Dynamic Techniques (PT, FCS, FRAP)

We consider three main fluorescence microscopy-related techniques as possible options to investigate drug delivery systems from different perspectives. Particle tracking (PT), fluorescence recovery after photobleaching (FRAP), and fluorescence correlation spectroscopy (FCS) are characterized by two main differences: (i) they monitor dynamics and diffusion in different ways (PT by determining full trajectories, FRAP by monitoring Fickian diffusion [71], and FCS by monitoring fluctuations due to Brownian motion); and (ii) they probe different detection areas (PT, full images; FRAP, defined regions of interest inside the images; and FCS, single laser spots). Therefore, these techniques provide access to different information, and in drug delivery research, they can be applied to different issues. Specifically, PT relies on the analysis of entire fluorescence microscopy images to rebuild the trajectories of diffusing moieties. It can be applied to relatively slow processes, and it requires complex data analysis; however, it allows for the determination of very complex dynamic processes (such as those occurring in cellular uptake). In FRAP, a region of interest is defined inside the image, where the fluorescent species are photobleached and their replacement upon diffusion from the neighboring regions (via Fickian diffusion) is then monitored and analyzed. It is a relatively simple technique that does not require complex experimental setup or data analysis; however, complex diffusive processes are generally difficult to disentangle, and relatively slow processes are generally considered. Therefore, FRAP is generally applied for diffusion issues that are restricted to lipid membranes, such as plasma membranes. Finally, in FCS the investigation area is limited to a single laser spot, where the fluctuation of the fluorescence intensity of fluorescent species—due to their Brownian motion—are analyzed. FCS requires a specific setup; however, it can probe very fast dynamics (such as the diffusion of a small molecule in water), can be applied to very diverse issues in drug delivery research (in the following, different examples will be presented), and requires less complex data analysis than PT—though it is less suited for disentangling highly complex diffusion modes.

The PT technique is based on the acquisition/analysis of multiple images of fluorescently labeled diffusing species, which are then analyzed to determine the trajectories of each diffusing species, and therefore analyze their dynamic behavior. In particular, by evaluating the displacement between the particle positions, it is possible to calculate the mean squared displacement (MSD) of particles linked to their diffusion coefficient. The dependence of the MSD on time provides information about the diffusion modes of the particles. A linear dependence of the MSD on time (*t*) is related to purely Brownian motion, where if the dependence of the MSD on time is ∝ *t^α^* with *α* < 1, then subdiffusive/cage/crowding effects occur, whereas with *α* > 1, active transport effects are present [72]. Therefore, PT can be profitably exploited to investigate the highly complex dynamics of DDSs in biological environments, particularly in the crossing of biological barriers [73]. Generally, PT can be performed with the standard widefield microscopes provided with fast cameras. However, the STED technique, coupled with resonant scanners (necessary to obtain relatively fast acquisition), can be valuable in allowing for the performance of PT with high spatial resolution. As previously discussed, DDSs loaded with drugs must cross biological membranes and enter cells to reach the target site and perform their biological task. Here, PT enables the direct detection and visualization of drug-loaded DDSs internalized in the cellular environments as well as the intracellular dynamics involved during the process. For instance, Chen et al. performed real-time imaging and particle tracking with confocal microscopy to study the endocytosis and intracellular trafficking of fluorescent polymer dots and carboxylate polystyrene nanoparticles, showing the kinetics of the process at the single-particle level [74,75,76]. Simultaneous and real-time monitoring of the motion and intracellular dynamics of mesoporous silica nanoparticles, labeled with pH-sensitive dyes, were reported by Mou et al., who performed single particle tracking in targeting lysosomes [77], while Tan et al. report on the successful tracking of the endocytic transport of aptamer-drug conjugates (ApDCs) in human cancer cell lines [78]. PT was also successfully employed to prove the successful uptake of tetrahedral DNA nanostructures in live cells [79]. One critical issue in nanoparticles’ delivery is represented by their size and surface modification, which dramatically influence the in vivo NPs’ fate. Klymchenko et al. exploit PT to analyze the diffusion of several dye-loaded poly(methyl methacrylate) nanoparticles with dimensions ranging from 7 to 50 nm. Their results revealed the existence of a critical limit of the size under which the free diffusion and spreading of the particles occur [67].

FRAP is based on the photobleaching of a region of interest (ROI) inside the sample through the employment of a high-intensity laser beam. A series of images is then acquired at normal illumination intensity to monitor the rate and extent of fluorescence intensity recovery inside the ROI. Specifically, from the rate of fluorescence intensity recovery (which is due to the replacement of the photobleached fluorescent dyes with active fluorescent species diffusing from the neighboring areas), the diffusion coefficient of the fluorescent species can be inferred; from the percentage of recovery compared to the theoretical fluorescence recovery, the mobile fraction of the probe is discriminated from the immobile fraction (see Figure 3b [69]) [80]. Overall, FRAP is generally applied to monitor slow processes, and particularly, to obtain information on the structure/viscosity of the medium where the fluorescent probe diffuses. In drug delivery research, FRAP has been profitably applied to characterize the retention properties of hydrogels designed from drug delivery applications [81], as well as to determine the interaction of nanocarriers with relevant biological barriers [82], such as their effect on membrane fluidity [83]. Being a well-established technique that was developed decades ago, it is recently gaining renewed interest thanks to the possibility of coupling it with STED. Recent FRAP–STED studies have been, for instance, applied for analyzing the intracellular dynamics of a dye released from a mesoporous silica nanocarrier [84], to discriminate the distribution of inner and outer nuclear proteins [85], and even to evaluate the diffusion processes in a living spine neck [86]—suggesting that, in the next few years, the FRAP–STED coupling could provide useful tools for DDS investigation that overcome the resolution limitations of FRAP.

FCS is a correlation technique similar to dynamic light scattering (which is commonly applied for DDS characterization and colloidal stability evaluation) [87]. The experimental setup is schematized in Figure 3c. Briefly, with a laser scanning confocal microscope, the laser beam is focused on a single spot of a sample containing the fluorescent species of interest; if the fluorescently labeled species are sufficiently diluted (typically, in the nM concentration range), then their fluorescence intensity significantly fluctuates with time, due to the diffusion of the species inside and outside of the excitation volume. These fluctuations are collected by a correlator to calculate the autocorrelation function of fluorescence intensity, which is the FCS output curve (two-color experiments can be also performed, resulting in a cross-correlation curve) [68,88]. From the analysis of FCS curves, much information can be obtained, specifically, the diffusion coefficient of the diffusing objects, their concentration, the dimensionality of the diffusing medium (i.e., 1D, 2D, or 3D), and their diffusion mode (i.e., bure Brownian or anomalous). Additionally, FCS measurements can be performed by pointing the laser in specific regions of a specimen; thus, at variance from DLS, FCS can probe the dynamics of the species of interest in specific localized areas—for instance, in different regions of a eukaryotic cell (see Figure 3b [16]). Thanks to the multiple opportunities offered by this technique, FCS has been employed in drug delivery research both in the characterization of DDSs and in the investigation of their behavior within biological fluids [89]. Concerning the characterization of DDSs, Salvatore et al. [90] monitored, through FCS, the formation of a multifunctional DDS made of magnetoliposomes decorated with therapeutic oligonucleotides. Specifically, by combining DLS with FCS (and, in particular, by exploiting the different nature of the techniques—the first one detecting scattering and the second one detecting fluorescence), it was possible to follow the different steps of the multifunctional DDS formation. In other studies, FCS was exploited to monitor the dimensionality of complex lipid architectures, designed as soft matter scaffolds for biomedical applications, to monitor the successful embedding of model drugs [91,92,93,94] or to determine their release [92,95]. In addition, FCS has also been profitably applied to investigate the dynamics of protein corona coating formation on nanoparticles [96,97]. In this respect, a major advantage of FCS (unlike DLS) is its inherent ability to monitor the dynamics of selected species (i.e., the fluorescently labeled ones), even in complex environments [98,99,100]. This property has been extensively exploited to monitor the behaviors of DDSs in biological environments. For instance, the sensitivity of FCS to concentration has been exploited to precisely determine the cellular uptake of cell-penetrating peptides [101]. Additionally, by exploiting the sensitivity of the technique for the diffusion coefficient of the fluorescent species of interest (and, therefore, on its size), it has been exploited to evaluate the form (i.e., assembled or disassembled) of the DDSs inside cells [102,103]. Recently, multiphoton FCS has been applied with fluorescently labeled polymeric nanoparticles in vivo to determine the flow inside the brains of mice, and at the same time, to determine the nanoparticles’ transport and degradation [104]. FCS is also a powerful tool for investigating dynamic processes involving cell membranes and biomimetic membranes [105,106]. Recently, STED has been coupled with FCS, allowing for the monitoring of the dynamics of fluorescent species in spots of reduced size (i.e., below 200 nm) [107,108]; this has been applied to the monitoring of lipid dynamics [109,110].

## 4. Conclusions

In the latest years, fluorescence microscopy-related techniques have exhibited continuous developments and progresses, both from technical and applicative perspectives, holding the promise to provide unprecedented tools for drug delivery research. In this review, we have revised the major fluorescence microscopy-related experimental techniques available for the characterization of drug delivery systems from static and dynamic points of view in different media, with a particular focus on the investigation within biological environments and in vivo. Indeed, the opportunities provided by fluorescence microscopy-related techniques to disentangle scientific issues typical of drug delivery research (spanning from the colloidal characterization of a DDS to its adhesion to biological membranes, its interaction with biomolecules, and its intracellular behavior) are countless and exponentially growing, allowing for the expectation that in the next few years the development of completely new tools and protocols will truly advance drug delivery research.
